# Relative contributions of adipose-resident CD146^+^ pericytes and CD34^+^ adventitial progenitor cells in bone tissue engineering

**DOI:** 10.1038/s41536-018-0063-2

**Published:** 2019-01-07

**Authors:** Yiyun Wang, Jiajia Xu, Leslie Chang, Carolyn A. Meyers, Lei Zhang, Kristen Broderick, Min Lee, Bruno Peault, Aaron W. James

**Affiliations:** 1 Department of Pathology, Johns Hopkins University, 21205 Baltimore, MD USA; 2 Department of Plastic Surgery, Johns Hopkins University, 21205 Baltimore, MD USA; 3 School of Dentistry, University of California, Los Angeles, 90095 CA USA; 4 UCLA and Orthopaedic Hospital Department of Orthopaedic Surgery, Orthopaedic Hospital Research Center, 90095 Los Angeles, CA USA; 5 Center For Cardiovascular Science and MRC Center for Regenerative Medicine, University of Edinburgh, Edinburgh, UK

## Abstract

Pericytes and other perivascular stem/stromal cells are of growing interest in the field of tissue engineering. A portion of perivascular cells are well recognized to have MSC (mesenchymal stem cell) characteristics, including multipotentiality, self-renewal, immunoregulatory functions, and diverse roles in tissue repair. Here, we investigate the differential but overlapping roles of two perivascular cell subsets in paracrine induction of bone repair. CD146^+^CD34^−^CD31^−^CD45^−^pericytes and CD34^+^CD146^−^CD31^−^CD45^−^adventitial cells were derived from human adipose tissue and applied alone or in combination to calvarial bone defects in mice. In vitro, osteogenic differentiation and tubulogenesis assays were performed using either fluorescence activated cell sorting-derived CD146^+^ pericytes or CD34^+^ adventitial cells. Results showed that CD146^+^ pericytes induced increased cord formation in vitro and angiogenesis in vivo in comparison with patient-matched CD34^+^ adventitial cells. In contrast, CD34^+^ adventitial cells demonstrated heightened paracrine-induced osteogenesis in vitro. When applied in a critical-size calvarial defect model in NOD/SCID mice, the combination treatment of CD146^+^ pericytes with CD34^+^ adventitial cells led to greater re-ossification than either cell type alone. In summary, adipose-derived CD146^+^ pericytes and CD34^+^ adventitial cells display functionally distinct yet overlapping and complementary roles in bone defect repair. Consequently, CD146^+^ pericytes and CD34^+^ adventitial cells may demonstrate synergistic bone healing when applied as a combination cellular therapy.

## Bone repair: synergistic healing from two types of fat cells

Different kinds of cells found surrounding blood vessels in fat play a complementary and synergistic role in bone healing. Aaron James from Johns Hopkins University in Baltimore, MD, USA, and colleagues derived two subsets of cells from human fat tissue: contractile cells known as pericytes that wrap around cellular lining of capillaries and tiny veins; and connective tissue cells known as adventitial cells that surrounds larger vessels. Under isolated culture conditions, pericytes stimulated the development of primitive blood vessels, whereas adventitial cells promoted early bone formation. The researchers applied the cells to the sites of bone defects in mice and saw that combined treatment with both pericytes and adventitial cells led to greater bone repair than treatment with either cell type alone.

## Introduction

The vascular wall within adipose tissue (AT) is a source of stromal progenitor cells, often referred to as perivascular stem/stromal cells (PSC), vascular wall-resident mesenchymal stem cell (MSC), or tissue-specific MSC. Perivascular cells have long been supposed to be the cell type culpable for pathologic vascular ossification.^1,2^ Perivascular AT is an appealing source of stromal cells for skeletal regenerative medicine, as it is an easily accessible and dispensable cell source.^3–5^ The unpurified stromal vascular fraction (SVF) of AT has been previously used for bone repair, but formed bone tissue unreliably^6^ or with a low efficacy.^7^ Variability in cell subset frequency within different preparations of SVF may represent one factor predisposing to unreliable tissue formation.

Cells within perivascular AT are well recognized to have MSC characteristics, including multipotentiality, self-renewal, immunoregulatory functions, and diverse roles in tissue repair. The in situ identification of pericytes as a tissue-resident MSC population was first reported in 2008,^8^ although the probable progenitor cell identity of pericytes had been shown as early as 1999.^9–11^ The identification of CD34^+^ progenitor cells within the tunica adventitia was described as early as 2007,^12,13^ and their tissue-resident MSC identity was most clearly documented in 2012.^14^ Both AT-derived CD146^+^ pericytes^8^ and CD34^+^ adventitial cells^14^ are multipotential when cultured under appropriate conditions (observed to form osteoblasts, chondroblasts, and adipocytes), and give rise to bone cells when implanted within^15^ or outside a bone microenvironment.^16^ Because of the overlapping features of CD146^+^ pericytes and CD34^+^ adventitial cells, they have most commonly been combined for tissue engineering applications under the umbrella term “perivascular stem/stromal cells, PSC” (see ref. ^17^ for a review).

Despite their shared perivascular residence, studies suggest that AT-derived CD146^+^ pericytes and CD34^+^ adventitial cells have clear differences. In earlier descriptions by Corselli et al., CD34^+^ adventitial cells can adopt a pericyte-like immunophenotype under appropriate culture conditions.^14^ This suggested a fluidity between perivascular cell types, but also that adventitial cells represent a more “stem” or progenitor cell type. Recent single-cell transcriptional analysis supports this concept of a functional and developmental hierarchy within the perivascular niche of human AT.^18^ Here, 178 individual perivascular cells from a single donor’s AT were examined on a Fluidigm platform. Among 429 gene transcripts examined, a clear separation between CD146^+^ pericytes and CD34^+^ adventitial cells was observed by hierarchical clustering and principal component analysis.^18^ Adventitial cells preferentially expressed a few genes of pluripotency or stemness (e.g., *NANOG, CMYC, KLF2*), growth factor receptors characteristic of progenitor cells (e.g., *FGFR2, PDGFRA, TGFBR1A*), as well as the osteogenic transcription factor *RUNX2*. In contrast, pericytes preferentially expressed genes involved in angiogenesis and vascular smooth muscle cell function (e.g., *ACTA2, ANGPT2*). In aggregate, these gene signatures suggested functionally relevant differences between CD146^+^ pericytes and CD34^+^ adventitial cells when placed in a bone defect microenvironment.

## Results

### Combinatorial efficacy with CD146^+^ pericytes and CD34^+^ adventitial cells in calvarial bone repair

Microvascular pericytes (CD146^+^CD34^−^CD31^−^CD45^−^) and adventitial progenitor cells (CD34^+^CD146^−^CD31^−^CD45^−^) were isolated from de-identified human white AT liposuction specimens based on previously established protocols.^19^ Fluorescence activated cell sorting (FACS)-based isolation of distinct perivascular cell populations is shown in Supplementary Figure 2. In brief, after removal of DAPI^+^ non-viable cells, CD31^+^ endothelium and CD45^+^ inflammatory cells, CD146^+^CD34^−^ pericytes represented 1.81% of total mononuclear cells, whereas CD34^+^CD146^−^adventitial cells represented 31.26% of total mononuclear cells. To discern the relative functions of each perivascular cell population, uncultured CD146^+^ pericytes and CD34^+^ adventitial cells were applied in equal numbers, alone or in combination, to non-healing calvarial bone defects in mice (see Supplementary Figure 1 and Supplementary Table 2 for a breakdown of cell numbers and treatment conditions). All cells were obtained from the same patient’s adipose sample. An osteoinductive hydroxyapatite-coated poly (lactic-co-glycolic acid) (PLGA) scaffold was used for cell delivery, as used in prior reports,^20^ and short-term incubation was performed prior to in vivo application. LIVE/DEAD cell viability staining was performed on selected scaffolds after cell seeding, and in all cases showed overall an even distribution of viable cells throughout the scaffold and negligible cell death across treatment groups (Supplementary Figure 3). Similar cell engraftment, similar distribution throughout the defect site, similar basal proliferative potential (Ki67 staining), and similarly infrequent apoptosis (terminal deoxynucleotidyl transferase (TdT) dUTP nick-end labeling; TUNEL staining) was observed among either CD146^+^ pericytes and CD34^+^ adventitial cells when examined at an early timepoint postoperative (3d postoperative, Supplementary Figure 4).

**Fig. 1:**
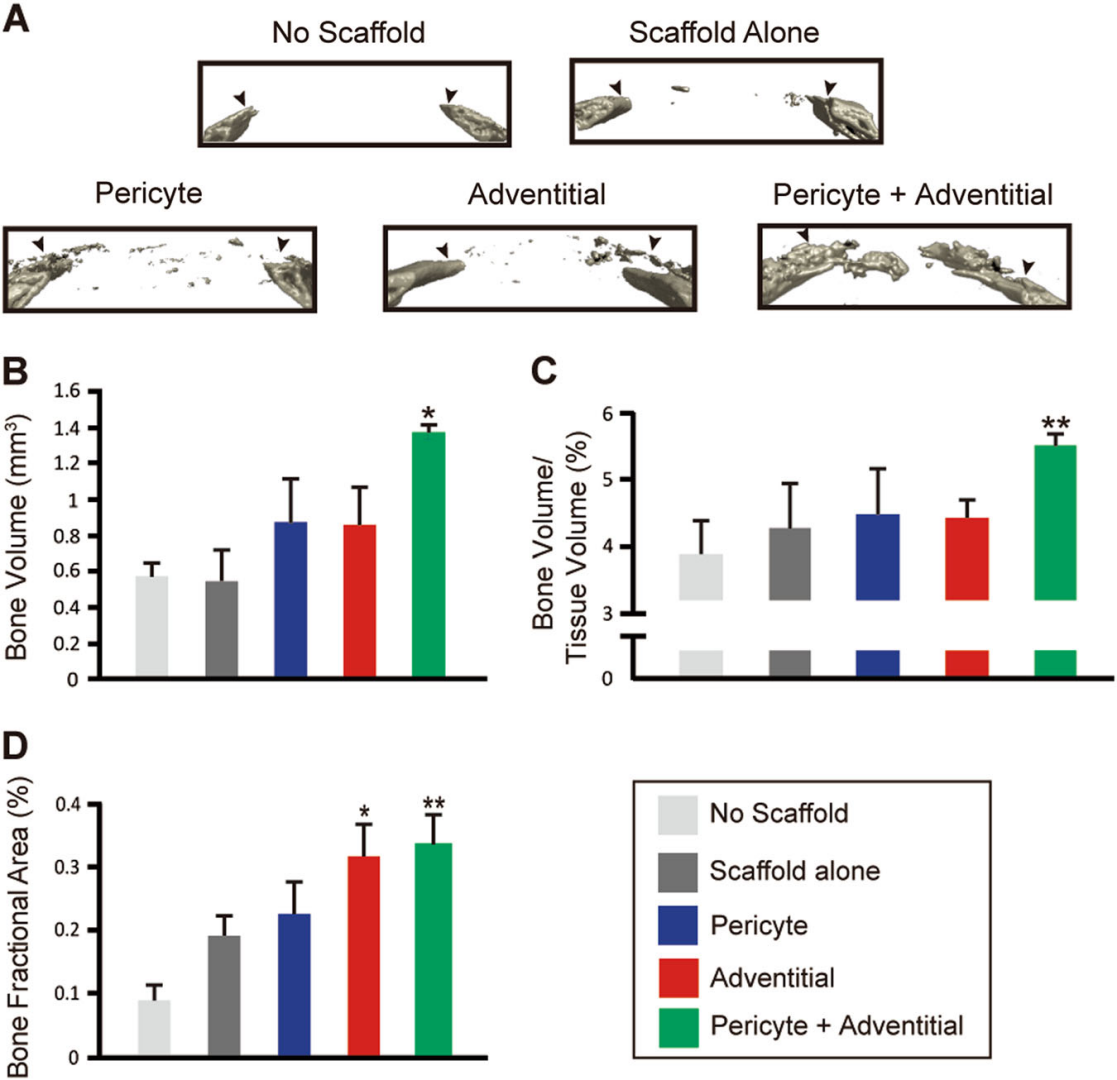
Microcomputed tomographic (microCT) imaging and analysis of calvarial defect healing with CD146^+^ pericytes or CD34^+^ adventitial cells, alone or in combination. **a** 3D coronal section reconstructions of calvarial defect site among “no scaffold” control, “scaffold alone“ control, or three different cell treatment groups. “Pericyte” group consisted of 2.5 × 10^5^ CD146^+^CD34^−^CD45^−^CD31^−^ pericytes. “Adventitial” group consisted of 2.5 × 10^5^ CD34^+^CD146^−^CD45^−^CD31^−^ adventitial cells. “Pericyte + Adventitial” group consisted of a 50%/50% split between each cell type (1.25 × 10^5^ adventitial cells and 1.25 × 10^5^ pericytes). **b**–**d** MicroCT quantification of bone repair in calvarial defects within each treatment group, 8 weeks postoperative. **b** Bone volume, **c** bone volume/ttissue volume, and **d** Bone fractional area. *P < 0.05, **P < 0.01 in comparison with “no scaffold” group. *N* = 46 bone defects per treatment group

Microcomputed tomography (microCT) imaging and analysis was performed at 8 weeks post implantation (Fig. 1). Images are shown in a 3D coronal cross-section (Fig. 1a), with quantitative analysis below (Fig. 1b–d). On coronal cross sections, the original defect edges are indicated by black arrowheads. As expected of a critical-size defect, the “no scaffold” group without treatment showed an essential absence of bone healing. Sparse radiodensity was observed with the “scaffold alone” without cell treatment. Patchy re-ossification was seen with either CD146^+^ pericytes or CD34^+^ adventitial cells alone. Interestingly, frank defect site re-ossification was only observed within the combination therapy of “pericytes + adventitial cells“. These findings were examined quantitatively, by analysis of bone volume (Fig. 1b, BV), fractional bone volume (Fig. 1c, bone volume/issue volume, BV/TV), and bone fractional area (Fig. 1d, BFA). In each case, the combination of equal numbers of pericytes + adventitial cells outpaced all other treatment groups in terms of indices of bone repair.

### Histologic appearance of CD146^+^ pericyte- and CD34^+^ adventitial cell-treated calvarial bone defects

**Fig. 2:**
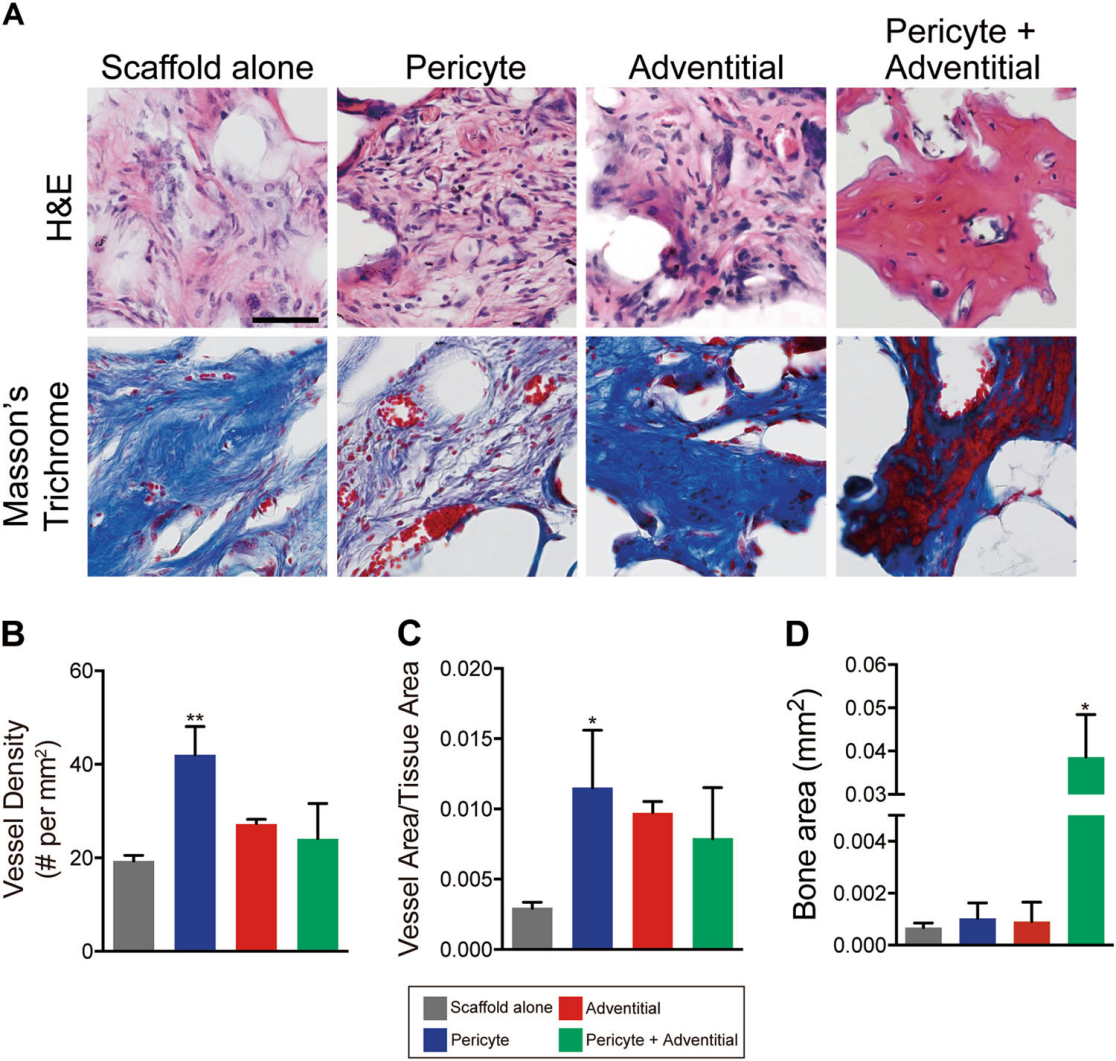
Histologic analysis of calvarial defect healing with CD146^+^ pericytes or CD34^+^ adventitial cells, alone or in combination. **a** Representative hematoxylin/eosin and Masson’s trichrome images of the defect site. Scale bar = 50 μm. Representative images are shown of the “scaffold alone” control, or three different cell treatment groups. “Pericyte” group consisted of 2.5 × 10^5^ CD146^+^CD34^−^CD45^−^CD31^−^pericytes. “Adventitial” group consisted of 2.510^5^ CD34^+^CD146^−^CD45^−^CD31^−^adventitial cells. “Pericyte + Adventitial” group consisted of a 50%/50% split between each cell type (1.25 × 10^5^ adventitial cells and 1.25 × 10^5^ pericytes). **b**, **c** Vascular histomorphometry of the defect site, including **b** blood vessel density, and **c** normalized vessel area per field. **d** Bone histomorphometry of the defect site, including bone area per field. *P < 0.05 in comparison with “Scaffold alone” group. *N* = 4−6 bone defects per treatment group. *N* = 6 images analyzed per sample

The histologic appearance of calvarial defect sites was next examined (Fig. 2). The “no scaffold” group had minimal tissue for examination and is not included in this analysis. Representative images from haemotoxylin and eosin (H&E) or Masson’s Trichrome-stained sections are shown (Fig. 2a). Sections of the “scaffold alone” treatment group showed scattered fibroblastic cells embedded in a fibrous stroma (Fig. 2a, far left). Sections of defect sites treated with CD146^+^ pericytes were observed to have a qualitative increase in small blood vessel density most easily observed on Trichrome-stained sections (Fig. 2a, middle left). Scattered immature woven bone formation was also seen (not shown). Sections of defect sites treated with CD34^+^ adventitial cells showed more conspicuous dense fibrous tissue along with a slight increase in defect site vascularization (Fig. 2a, middle right). Conspicuous bone formation was only observed among the combination cellular therapy “pericytes + adventitial” cells (Fig. 2a, far right). These observed differences were next quantified by histomorphometric analysis of serial cross sections (Fig. 2b–d). A trend toward increase defect site vascularization was observed across all cell therapies, as assessed by both blood vessel density and vessel area (Fig. 2b, c). These differences were most robust and achieved statistical significance among the “pericyte only” cell treatment group, which demonstrated a 117% increase in vessel density (Fig. 2b, blue bars) and 287% increase in normalized vessel area (Fig. 2c, blue bars). Quantification of bone matrix was next performed. In line with our histologic observations, a significant increase in bone area was only appreciated with the combination therapy “pericytes + adventitial” cells (Fig. 2d, green bars). Finally, we assessed for long term persistence of human perivascular cells within the defect site. Consistent with prior reports suggesting a primary paracrine mechanism of action for human PSC,^21^ immunohistochemistry against human nuclear antigen showed no significant persistence of human cells at the eight week study endpoint (Supplementary Figure 5).

### Vascular tubulogenesis stimulated by CD146^+^ pericytes or CD34^+^ adventitial cells

**Fig. 3:**
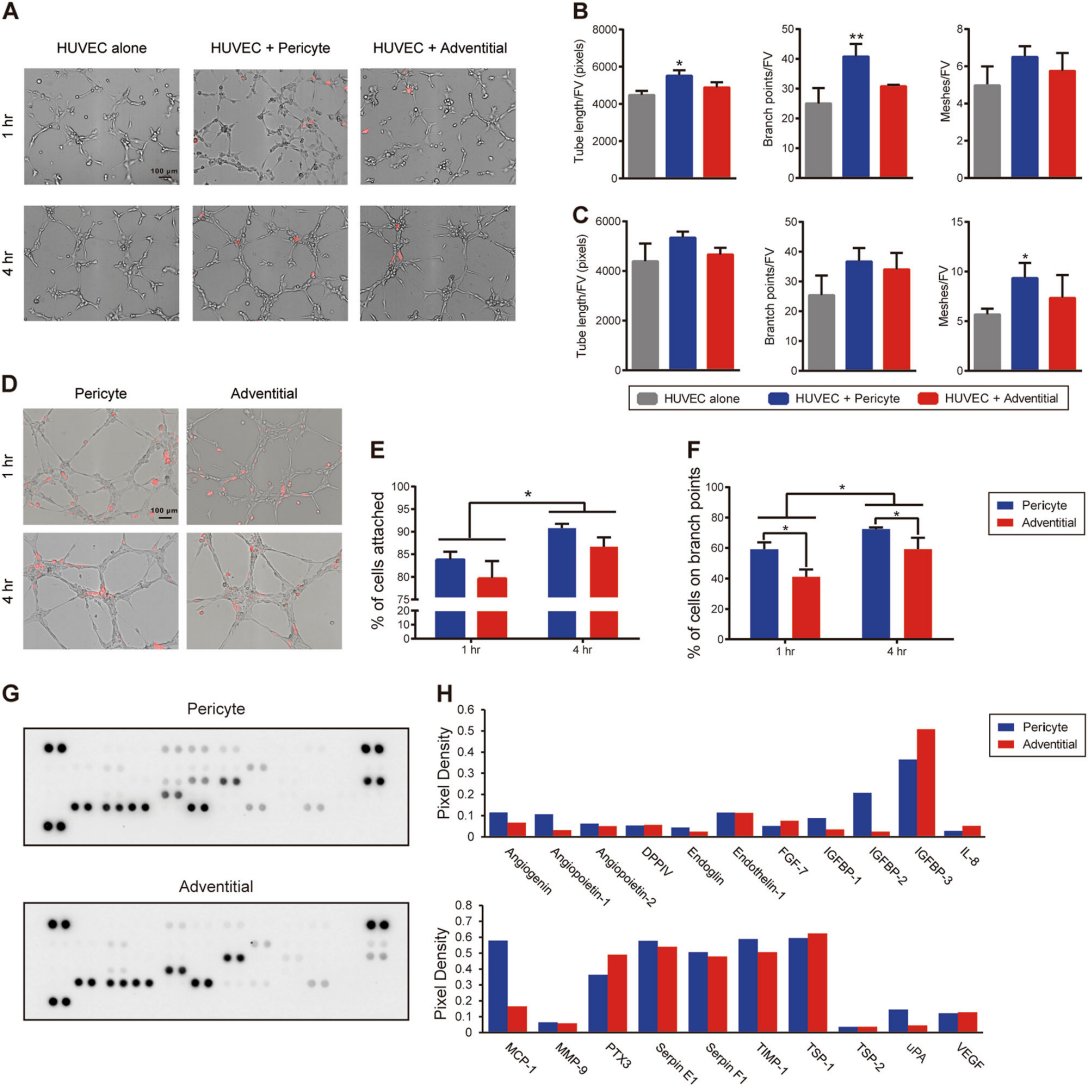
Differential effects on HUVEC cord formation by CD146^+^ pericytes or CD34^+^ adventitial cells. **a**, **b** HUVECs were seeded in culture alone, or with either CD146^+^ pericytes or CD34^+^ adventitial cells. In all cases, pericytes or adventitial cells were labeled with PKH26^+^ red fluorescent lipophilic dye. Cord formation was assessed at 1 and 4 h post seeding. **a** Representative images at × 10magnification. Left: HUVECs cultured alone (HUVEC alone). Middle: HUVECs and CD146^+^CD34^-^CD45^−^CD31^−^ pericyte co-culture (HUVEC + Pericyte). Right: HUVECs and CD34^+^CD146^−^CD45^−^CD31^−^adventitial cells co-culture (HUVEC + Adventitial). **b**, **c** Quantification of length, branchpoints, and meshes per field view (FV) of **a** at **b** 1 h and **c** 4 h. *P < 0.05, **P < 0.01 in comparison with HUVEC alone group. **d** Relative attachment of CD146^+^ pericytes or CD34^+^ adventitial cells on pre-formed HUVEC cords. PKH26^+^ red fluorescent-labeled pericytes or adventitial cells were seeded on newly formed HUVEC network. Images shown at 1 and 4 h after perivascular cell seeding. Both pericytes and adventitial cells showed significant tropism for newly formed HUVEC network. **e** Quantification of attachment of cells on the networks, represented as a percentage of the PKH26^+^ cells in contact with the network per total PKH26^+^ cells per field. **f** Quantification of attachment of cells on the branchpoints, represented as a percentage of PKH26^+^ cells in contact with the branchpoints per total PKH26^+^ cells per field. *P < 0.05. *N* = 3 wells and three images per group. **g** Angiogenic protein array demonstrating secreted proteins from either CD146^+^ pericytes (above) or CD34^+^ adventitial cells (below) after 24 h in culture. Captured antibodies to specific target proteins were spotted in duplicate. **h** Secretion level of each angiogenesis-related protein measured as pixel density of the pair of duplicate spots and normalized to negative control spots

In order to gain a more isolated understanding for the functional differences between CD146^+^ pericytes and CD34^+^ adventitial cells, co-culture experiments were first performed with human umbilical vein endothelial cells (HUVECs) (Fig. 3). CD146^+^ pericytes or CD34^+^ adventitial cells were labeled with PKH26 dye and cultured with HUVEC at equal ratios. Cord formation was assessed at 1 and 4 h post seeding (Fig. 3a–c). Results showed that CD146^+^ pericytes induced a significant increase in cord formation at both 1 and 4 h, as assessed by random imaging fields (Fig. 3a). Quantification of cord length, branchpoints, and mesh number was assessed per field of view (FV) (Fig. 3b, c). CD146^+^ pericytes induced an increase in all metrics across all timepoints, although not all datapoints reached statistical significance (blue versus gray bars, Fig. 3b, c). In contrast, no statistically significant difference in cord formation was appreciated with CD34^+^ adventitial cell co-culture in comparison with HUVECs alone (red versus gray bars, Fig. 3b, c).

Next, we inquired as to whether CD146^+^ pericytes and CD34^+^ adventitial cells had differential tropism to HUVEC networks in vitro (Fig. 3d–f). Here, CD146^+^ pericytes or CD34^+^ adventitial cells were seeded on pre-formed HUVEC networks. The frequency of attachment of PKH26^+^ perivascular cells to cords (Fig. 3e) or cord branchpoints (Fig. 3f) was assessed at early timepoints post seeding. Qualitatively, both pericytes and adventitial cells showed prominent tropism to the outside aspects of cords (Fig. 3d). The percentage attachment was assessed at 1 and 4 h post seeding (Fig. 3e, f). The majority of both perivascular cells showed cord attachment, which significantly increased over time (Fig. 3e, 80–85% attachment at 1 h, 87–91% attachment at 4 h). A trend toward increased CD146^+^ pericyte attachment was noted, although this did not reach statistical significance at either timepoint. CD146^+^ pericytes were also noted to preferentially attach to the branchpoints of networks (see especially 4 h images post seeding). These findings were quantified, demonstrating a significant preference for CD146^+^ pericytes rather than CD34^+^ adventitial cells to attach to branchpoints (Fig. 3f, 70–74% pericyte attachment to branchpoints, in comparison with 50–65% adventitial cell attachment to branchpoints). Thus, in similarity to our in vivo findings, CD146^+^ pericytes appear to preferentially induce cord formation in vitro. In addition, although tropism toward vascular cords is a conserved feature of both perivascular cell populations, CD146^+^ pericytes appear to preferentially be found at sites of cord branching.

In order to begin to understand the differences in paracrine-induced cord formation between CD146^+^ pericytes and CD34^+^ adventitial cells, the angiogenic secretome of patient-identical CD146^+^ pericytes vs CD34^+^ adventitial cells was next examined (Fig. 3g, h). Of 55 angiogenesis-related proteins, 21 showed detectable expression in the supernatant of either pericytes or adventitial cells (Fig. 3g). Increased pericyte secretion of five proteins were observed, including MCP1 (C-C motif chemokine ligand 2), IGFBP1 (Insulin like growth factor binding protein 1), IGFBP2 (Insulin-like growth factor binding protein 1), ANGPT1 (Angiopoietin 1), and uPA (Plasminogen activator, urokinase) (Fig. 3h). Conversely, insulin-like growth factor binding protein 3 and Pentraxin 3 showed higher secretion among adventitial cells. Interestingly, no difference in vascular endothelial growth factor was detected between CD146^+^ pericytes with CD34^+^ adventitial cells.

### Paracrine-induced osteogenesis by CD146^+^ pericytes or CD34^+^ adventitial cells

**Fig. 4:**
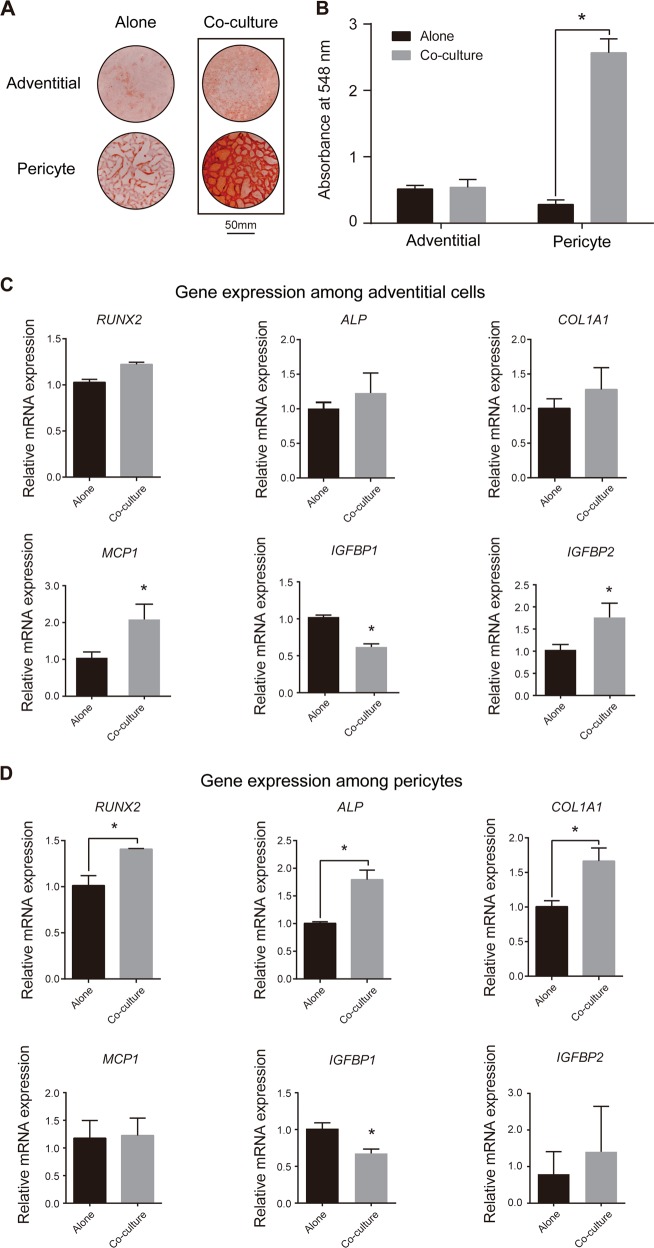
Bidirectional paracrine effects of CD146^+^ pericytes and CD34^+^ adventitial cells during osteogenic differentiation in non-contact co-culture in vitro. **a** Representative Alizarin Red S staining, whole well images after 14 days osteogenic differentiation. **b** Quantitative analysis of Alizarin Red S staining shown using absorbance at 548 nm. **c** Quantitative RT-PCR analysis of osteogenic gene markers (*RUNX2, ALP, COL1A1*) and angiogenesis-related gene markers (*MCP1, IGFBP1, IGFBP2*) among CD34^+^ adventitial cells with or without pericyte co-culture, day 7 of osteogenic differentiation. **d** Quantitative RT-PCR analysis of osteogenic and angiogenic gene markers among CD146^+^ pericytes with or without adventitial cell co-culture, day 7 of osteogenic differentiation. *P < 0.05. *N* = 3 wells per condition

Our in vivo studies suggested potential combinatorial efficacy with CD146^+^ pericytes and CD34^+^ adventitial cells in bone-defect healing. Beyond differences in stimulating vascularization, we next sought to identify potential functional differences paracrine-induced osteogenic differentiation (Fig. 4). Here, the potential interaction between CD146^+^ pericytes and CD34^+^ adventitial cells in speeding osteogenic differentiation of the counterpart cell type was examined. CD146^+^ pericytes and CD34^+^ adventitial cells were placed in non-contact co-culture, and osteogenic differentiation was assessed under standard osteogenic differentiation conditions. All cells were obtained from the same patient’s adipose sample. Alizarin red staining of bone nodules was performed at 14 days after co-culture (Fig. 4a, b). Staining and photometric quantification showed that CD34^+^ adventitial cells induced a robust increase in bone nodule formation among CD146^+^ pericytes. Conversely, CD146^+^ pericytes showed no significant effect on the osteogenic differentiation of their counterpart CD34^+^ adventitial cells. These findings were recapitulated by gene expression analysis by quantitative real-time polymerase chain reaction (RT-PCR) (Fig. 4c, d). Little change in osteogenic gene expression was observed among CD34^+^ adventitial cells with or without paracrine stimulation by CD146^+^ pericytes. Instead, changes in expression of angiogenesis-related factors were identified, including *MCP1, IGFBP1*, and *IGFBP2* (Fig. 4c). In contrast, increased transcript abundance for the osteogenic transcription factor *RUNX2* (*Runt-related transcription factor 2*), enzyme *ALP* (*Alkaline Phosphatase*), and matrix component *COL1A1* (*Type I Collagen*) were all found by paracrine stimulation of pericytes by CD34^+^ adventitial cells (Fig. 4d). In contrast, adventitial cells induced little change in angiogenesis-related gene expression among CD146^+^ pericytes (Fig. 4d). In sum, these in vitro findings suggest functional differences between perivascular subpopulations. Paracrine-induced vasculogenesis is most appreciable among CD146^+^ pericytes, whereas paracrine-induced osteogenesis is most associated with CD34^+^ adventitial cells.

## Discussion

Bone healing is associated with a confluence of factors including hemorrhage, local inflammation, progenitor cell migration, osteogenic and chondrogenic differentiation, and vascularization, among other local and systemic factors. The present study examined in detail the effects of perivascular progenitor cell subpopulations on just two of these factors: osteogenic differentiation and defect vascularization. In summary, we observed that subpopulations of perivascular progenitor cell types demonstrate distinct, overlapping, and potentially synergistic effects on osteogenesis and vasculogenesis during bone repair. CD146^+^ pericytes have a more prominent pro-vasculogenic effect, accompanied by increased elaboration of pro-angiogenic factors such as ANGPT1 and uPA. Given the pericyte’s native residence and known roles in regulating angiogenesis and revascularization,^22^ these findings are to some extent intuitive. In contrast, CD34^+^ adventitial cells have more prominent paracrine pro-osteogenic effects. Given these distinct but overlapping roles in bone repair, future use of a combination progenitor cell therapy may be a more efficacious approach than use of a single, more homogenous cell preparation. Although equal numbers of pericytes and adventitial cells have been mixed in the combination progenitor cell therapy experiments reported here, it will be important to determine whether different ratios of these two cell types can support even more dramatic osteogenesis. As well, our observation that CD146^+^ pericytes induce heightened vascularization suggests that this population may be most useful alone in contexts of ischemic tissue repair. This concept is borne out by prior studies, in which the promise of pericyte application in cardiac ischemic repair has been well-demonstrated.^22–25^

Previous investigators have used cell separation techniques including FACS to purify AT-derived cell populations for tissue engineering purposes with some success.^26–29^ In fact, AT-derived progenitor cells with a predilection for bone,^26^ adipose,^27^ or cartilage formation^27^ have been defined. However, existing studies segregate AT progenitor cell therapies based on differential expression of cell surface signaling receptors, such as TGF-β co-receptors^26,27^ or BMP receptors.^28,29^ This approach is fundamentally distinct from that presented here, and is an attempt to retrospectively curate a mixed cell population rather than prospectively isolate a cell type of interest. At present, it is not clear how prior cell preparations overlap or correlate to either CD146^+^ pericytes or CD34^+^ adventitial cells. Single-cell analysis of AT-derived pericytes and adventitial cells did not find these previously used antigens as among those most differentially expressed between perivascular cell types (including *CD90, CD105, BMPR1A*, or *BMPR1B*).^18^

Several caveats exist toward the broader extrapolation of these results. First, the bone model chosen is an intramembranous model of bone repair and was chosen for its ease and reproducibility. It remains to be seen if CD146^+^ pericytes and CD34^+^ adventitial cells have similar combinatorial efficacy exists in models of endochondral bone repair. Prior studies suggest that CD146^+^ pericytes and CD34^+^ adventitial cells do indeed have differential paracrine functions in chondrogenic differentiation in vitro.^30^ Zhang et al. observed that CD34^+^ adventitial cells may support chondrocyte proliferation, whereas CD146^+^ pericytes stimulate glycosaminoglycan and cartilage-associated collagen production.^30^ Second, it is clear from both histologic, structural, and transcriptional standpoints that both perivascular populations harbor intrinsic cellular diversity.^31^ Pericytes can be subdivided across a spectrum of small vessel types including arterioles, capillaries, and venules. In mice, functionally distinct pericyte subtypes have been examined using nestin and NG2 transgenic reporters.^32–34^ Similarly and in human tissue, Hardy et al. showed that aldehyde dehydrogenase bright and dim subpopulations of CD146^+^ AT-derived pericytes were transcriptionally distinct, and this marker could be used to identify a more “primitive” pericyte subpopulation.^18^ CD34^+^ adventitial cells can likewise be categorized based on vessel type between arteries and veins. Gli1^+^ adventitial cells in mice have been shown to harbor progenitor cell characteristics and give rise to the tunica media in physiologic and pathologic conditions.^35^ Nevertheless, our own unpublished observations suggest that CD34 highlights a broader perivascular cell population, and that Gli1^+^ adventitial cells represent only a subpopulation within this more largely distributed CD34^+^ perivasculature.

In summary, perivascular cell preparations from AT are an appealing autologous cellular therapy for bone repair. CD146^+^ pericytes and CD34^+^ adventitial cells share fundamental attributes including progenitor cell identity, multipotentiality, and immunoregulatory features. Yet, functional differences exist in paracrine-induced osteogenesis, vasculogenesis, and paracrine-induced bone repair between perivascular progenitor cell populations. For the future, detailed studies that improve upon our correlation between organ and vessel of origin, distinguishing cell surface markers, and functional outcome post-transplantation are required to better harness the regenerative properties of the perivasculature.

## Methods

### Isolation of human CD146^+^ pericytes and CD34^+^ adventitial cells

Human lipoaspirate was obtained from healthy adult donors under institutional IRB approval with a waiver of informed consent, and was stored no > 72 h at 4 °C before processing. No patient identifiers were obtained. The SVF was obtained by collagenase digestion, according to previously published methods.^19^ In brief, lipoaspirate was washed with an equal volume of phosphate-buffered saline (PBS). The washed lipoaspirate was digested with 1 mg/ml type II collagenase in Dulbecco’s Modified Eagle’s Medium (DMEM) containing 3.5% bovine serum albumin (Sigma-Aldrich, St. Louis, MO) at 37 °C for 70 min under agitation. Adipose cells were isolated and removed by centrifugation. The cell pellet was resuspended and incubated in red cell lysis buffer (155 mm NH4Cl, 10 mm KHCO3, and 0.1 mm ethylenediaminetetraacetic acid (EDTA) at room temperature for 10 min. After centrifugation, the cells were resuspended in PBS and filtered at 70 μm. The resulting SVF was further processed for FACS. Cells were incubated with anti-CD34^−^ allophycocyanin (1:100; BD Pharmingen, San Diego, CA), anti-CD146-fluorescein isothiocyanate (1:100; Bio-Rad, Hercules, CA), anti-CD45^−^ allophycocyanin-cyanin 7 (1:100; BD Pharmingen) and anti-CD31^−^ allophycocyanin–cyanin 7 (1:100, Bio Legend, San Diego, CA) for 20 min on ice. See Supplementary Table 1 for a list of antibodies used. In this manner, pericytes (CD146^+^CD34^−^CD45^−^CD31^−^) and adventitial cells (CD34^+^CD146^−^CD45^−^CD31^−^) were isolated. Data were collected on a MoFlo XDP (Beckman Coulter, Indiana, USA) and analyzed using Summit Software. For in vitro studies, cells were expanded at 37 °C in a humidified atmosphere containing 95% air and 5% CO2. Unless otherwise stated, cells were cultured in DMEM containing 20% fetal bovine serum (FBS), and 1% penicillin/streptomycin.

### Mouse calvarial defects

All animal studies were performed with institutional ACUC approval within Johns Hopkins University, complying with all relevant ethical regulations. Non-healing, critical-sized (4 mm diameter) calvarial defects were performed in the parietal bone of 18-week-old male NOD/SCID mice as previously described (001303, Jackson Labs, CA, USA).^36^ After anesthesia with isoflurane and oxygen mixture, subcutaneous injection of buprenorphine (0.05 mg/kg) was used for pain relief. The hair over the cranial bone was shaved and the skin was aseptically prepared using poviodine/betadine scrub. A sagittal skin incision was made and the pericranium was removed from the parietal bone. A 4 mm full-thickness defect was made using a high-speed dental surgical drilling unit with a trephine, without injury to the adjacent sutures or underlying dura mater. After flushing the defect site with normal saline, a custom fabricated, previously validated, hydroxyapatite-coated 85/15 PLGA scaffold was placed (PLGA inherent viscosity 0.55–0.75 dL/g). In brief, for scaffold fabrication, PLGA/chloroform solutions were mixed with 200–300 m diameter sucrose to obtain 92% porosity (volume fraction), and compressed into thin sheets in a Teflon mold. A freeze-drying overnight, scaffolds were immersed in three changes of ddH_2_0 to dissolve the sucrose, and gently removed from the Teflon plate with a fine-tip spatula. Next, apatite-coating solution was prepared and applied to PLGA scaffolds as described in previous studies.^37^ Scaffolds were subjected to glow discharge argon plasma etching, followed by serial incubation in simulated body fluids.^37^ The apatite-coated scaffolds were washed with sterile ddH_2_0 to remove excess ions and lyophilized before further studies. See ref. ^20^ for further a description of scaffold fabrication.

Animals were divided into five treatment groups: (1) No scaffold group (an empty defect), (2) Scaffold alone group (PBS without cells placed on scaffold), (3) Pericyte group (2.5×10^^5^ CD146^+^CD34^−^CD45^−^CD31^−^pericytes in PBS were incubated with the scaffold for 3 h and then placed in the defect site), (4) Adventitial group (2.5×10^5^ CD34^+^CD146^−^CD45^−^CD31^−^adventitial cells were incubated with the scaffold for 3 h and then placed in the defect site), and (5) Pericyte + Adventitial group: (1.25×10^5^ adventitial cells and 1.25×10^5^ pericytes were incubated with the scaffold for 3 h and then placed in the defect site). Total cell numbers were based on prior publications.^19^ See Supplementary Figure 1 and Supplementary Table 2 for treatment group allocation. Finally, the skin was sutured closed. Defects were harvested at 8 weeks after surgery for analysis.

### Validation of scaffold seeding

Pericytes or adventitial cells alone (2.5×10^5^ cells, *n* = 3 per group) were incubated with scaffold using the above protocol for 3 h and then visualized using the Live/Dead kit according to the manufacturer’s instructions (Thermo fisher Scientific, MA, USA). The cellular distribution as well as Live/Dead staining within the scaffold was visualized in *n* = 6 random high power fields per scaffold by confocal microscopy with z-stack image acquisition (Zeiss LSM 800, Germany).

### MicroCT imaging and analysis

Calvaria were dissected free and evaluated using a SkyScan1175 (Bruker, MA, USA) high-resolution microCT imaging system. Each calvaria was scanned separately at 65 kV and 153 µA with a 1.0-mm aluminum filter to obtain a 10 µm voxel size. Scan slices were acquired in the coronal plane by placing the bone parallel to the *z*-axis of the scanner. NRecon (Bruker) was used to reconstruct images using the following settings: smoothing 1, ring artifact reduction of 5% and beam hardening correction of 20%, and quantitative analysis was performed using CTAn (Bruker) in accordance with the recommendations of the American Society for Bone and Mineral Research.^38^ Volumes of interest were disc shaped to encompass the circular bone defect and were 4.3 mm in diameter. All analysis was performed in a blinded fashion.

### Histology and histomorphometry

Eight weeks postoperatively, calvaria of mice were harvested, fixed in 4% paraformaldehyde for 24 h, decalcified with 12.5% EDTA (pH7.0) for 30 days, and embedded in paraffin. Calvarial defect sections were obtained in a coronal plane at 10 um thickness. Immunohistochemistry for Human Nuclear Antigen was performed using the primary antibody against human nuclei (MAB1281, Millipore, CA, USA), a biotinylated secondary antibody (BA-9200, Vector Labs, CA, USA), and the chromogenic DAB Peroxidase Substrate Kit (SK-4100, Vector Labs, CA, USA) according to the manufacturer instructions. Sections were stained with H&E or Masson’s Trichrome (TH15, Sigma-Aldrich, MO, USA). Random 10 × and 20 × images of H&E-stained slides (*N* = 6 images per sample) were obtained for semi-quantitative histomorphometric analysis, including the following parameters: (1) bone area (mean area measurement of bone expressed in mm^2^), (2) vessel density (number of vessels per mm^2^ tissue area), (3) vessel area (total vascular area per tissue area). All analysis was performed in a blinded fashion.

For PKH26 (PKH26GL, Sigma-Aldrich, MO, USA) visualization in vivo, cells were labeled with PKH26 fluorescent dye according to the manufacturer’s instructions, followed by engraftment in the calvarial defect model. Three days postoperatively, calvaria of mice were harvested, fixed in 4% paraformaldehyde for 24 h, decalcified with 12.5% EDTA (pH7.0) for 30 days, and embedded in OCT. Calvarial defect sections were obtained in a coronal plane at 15 µm thickness. In vivo cell engraftment/persistence was examined by fluorescence microscopy (Leica DM6, Leica Microsystems, Wetzlar, Germany). TUNEL staining (C10617, Invitrogen, MA, USA) was conducted within the sections according to the manufacturer’s instructions. For Ki67 immunofluorescence, sections were washed in 1× PBS, blocked in 5% normal goat serum (S-10000, Vector Labs, CA, USA), and incubated with primary antibodies specific for Ki67 (1:200, ab16667, Abcam, Cambridge, MA) at 4 ℃ overnight. Then sections were incubated with Alexa Fluor® 647-conjugated Secondary antibodies (1:200, ab150083, Abcam, Cambridge, MA), and mounted with mounting medium containing DAPI (H-1500, Vector Labs, CA, USA).

### Cord formation assays

CD146^+^ pericytes and CD34^+^ adventitial cells derived from the same patient and of equal passage number were labeled with PKH26 red fluorescent dye before use (PKH26GL, Sigma-Aldrich, MO, USA). Growth factor reduced Matrigel (BD Biosciences, CA, USA) was plated in 96-well culture plates and incubated at 37 °C to polymerize for 30 min.

For co-culture assays, HUVECs (C2517A, Lonza, USA) (15,000 cells/well) were seeded at the same time with either CD146^+^ pericytes or CD34^+^ adventitial cells (500 cells/well) on the polymerized matrigel. Cells were cultured with EGM2: DMEM (1:1) for 1 h. Cord formation was observed by microscopy by analysis of random × 10 fields (IX71, Olympus, Japan), including cord length per FV, branchpoints, per PV, and mesh numbers per FV. *N* = 3 wells and three images per group.

To monitor cell attachment to already formed vascular cords, HUVECs (15,000 cells/well) were seeded on the polymerized matrigel for 3 h for cord formation. Then PKH26-labeled pericytes or adventitial cells (3500 cells/well) were added to wells containing HUVEC cords and cultured for another 1 and 4 h. The attachment of CD146^+^ pericytes and CD34^+^ adventitial cells on HUVEC-generated cords was observed by analysis of random 10x fields. Attachment was defined by two methods, including (1) cells attached and spread on the HUVEC cords, or (2) cells attached to branchpoints. *N* = 3 wells and three images per group.

### Secretome analysis

CD146^+^ pericytes and CD34^+^ adventitial cells derived from the same patient and of equal passage number were plated at 1.5×10^5^/ml density in six-well plate cell culture dishes (2 mL per well). Upon subconfluency, standard growth medium was substituted for serum free DMEM (1.5 mL per well). Supernatant was collected 24 h later, centrifuged at 500 g for 5 min and then stored at − 20℃. Secretome analysis was performed with the Human Angiogenesis Array kit (ARY007) according to the manufacturer’s instructions (R&D system, Minneapolis, MN, USA). Visualization was performed with the ChemiDoc Touch Imaging System (Bio-Rad, CA, USA). The average signal (pixel density) of the pair of duplicate spots representing each angiogenesis-related protein was normalized on an averaged background signal from negative control spots. Corresponding signals were compared to determine the relative difference in angiogenesis-related proteins secreted by CD146^+^ pericytes and CD34^+^ adventitial cells.

### Osteogenic differentiation assays

CD146^+^ pericytes and CD34^+^ adventitial cells derived from the same patient sample were cultured in osteogenic differentiation medium, composed of DMEM, 10% FBS, 1% penicillin/streptomycin with 100 nm Dexamethasone, 10 mm β-glycerophosphate, and 50 μm ascorbic acid (Sigma-Aldrich). Medium was changed every 3 days. For non-contact co-culture, 24-well Transwell plates (Millipore, Darmstadt, Germany) with 0.4 μm pore filters were used. A total of 1 × 10^5^/ well of either CD146^+^ pericytes or CD34^+^ adventitial cells were seeded in 24-well plates on the lower chamber. The opposing cell type was seeded on the upper transwell plates (1 × 10^4^/well).

To detect mineralization, cultures were stained with Alizarin Red S at 14 days of differentiation (Sigma-Aldrich, USA). Finally, 0.1 n sodium hydroxide was used to dissolve the calcium precipitate and the solving liquid was quantified by absorbance at 548 nm (Epoch spectrophotometer, Biotek, VT, USA). *N* = 3 wells per condition.

To analyze osteogenic gene and angiogenic-related gene expression, TRIzol reagent (Life technology, CA, USA) was used for total RNA isolation at 7 days of differentiation. RNA was reverse transcribed into cDNA by iScript cDNA Synthesis Kit (Bio-Rad, CA, USA) according to the manufacturer’s instructions. RT-PCR was performed using SYBR Green PCR Master Mix (Thermo fisher Scientific, MA, USA). Primer information presented in Supplementary Table 3.

### Statistical analysis

Results are expressed as the mean ± SD. Statistical analysis was performed using either a Student’s *t* test for a two sample comparison, or analysis of variance followed by a post hoc Student’s *t* test (Graphpad Software 6.0). *P < 0.05 and **P < 0.01 were considered significant.

## Supplementary information

SUPPLEMENTARY MATERIAL

## Data Availability

The data sets generated during and/or analyzed during the current study are available from the corresponding author on reasonable request.

## **Supplementary information**

**Supplementary information** accompanies the paper on the *npj Regenerative Medicine* website (10.1038/s41536-018-0063-2).
