# Supplementary material for: Relative contributions of adipose-resident CD146+ pericytes and CD34+ adventitial progenitor cells in bone tissue engineering
Source: NPJ Regen Med. 2019 Jan 7;4:1. doi: 10.1038/s41536-018-0063-2 (PMC6323123; doi:10.1038/s41536-018-0063-2)
Supplement: Supplementary file 1 — SUPPLEMENTARY MATERIAL [file 41536_2018_63_MOESM1_ESM.pdf]

Supplementary Data

Supplementary Figures and Figure Legends

Supplementary Figure 1. Schematic of cell isolation and animal treatment groups. (Above) Human lipoaspirate is processed with a collagenase digestion to yield the stromal vascular fraction (SVF), which is further partitioned to identify CD146+CD34-CD45-CD31- pericytes and CD34+CD146-CD45-CD31- adventitial cells. (Below) Cells were placed in equal numbers in a 4mm circular parietal bone defect in NOD scid mice. Cell numbers and treatment conditions are outlined (see also **Supplementary Table 2**).

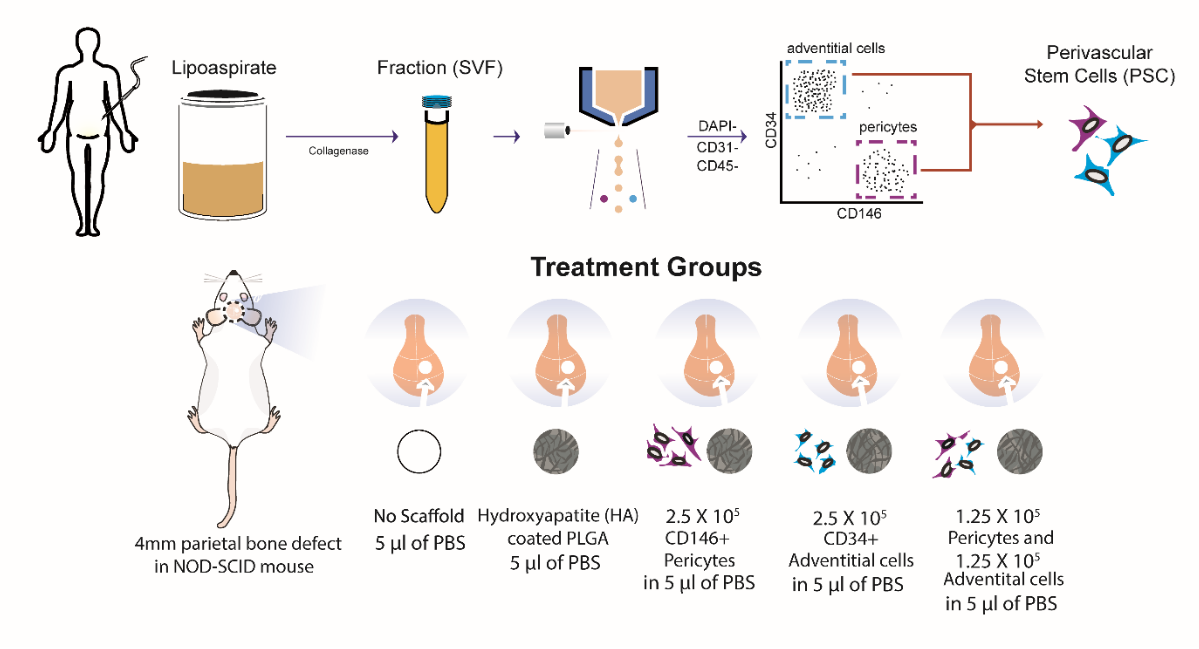

Supplementary Figure 2. Gating strategy to sort CD146$^+$ pericyte (CD31$^-$CD45$^-$CD146$^+$) and CD34$^+$ adventitial cell (CD31$^-$CD45$^-$CD34$^+$). Shown is the cell preparation used for *in vivo* application.

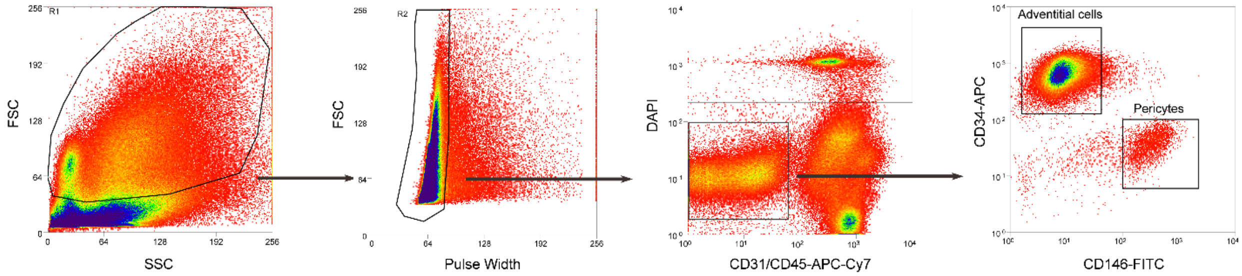

Supplementary Figure 3. CD146$^+$ pericytes or CD34$^+$ adventitial cells were labeled with the Live/Dead kit after incubation with the HA-PLGA scaffold for 3 hours. Cells were dispersed within the pores of the PLGA scaffold, with high viability across both cell types. Live cells appear green (Ex/Em = 488/509 nm), while dead cells appear red (Ex/Em = 545/617 nm).

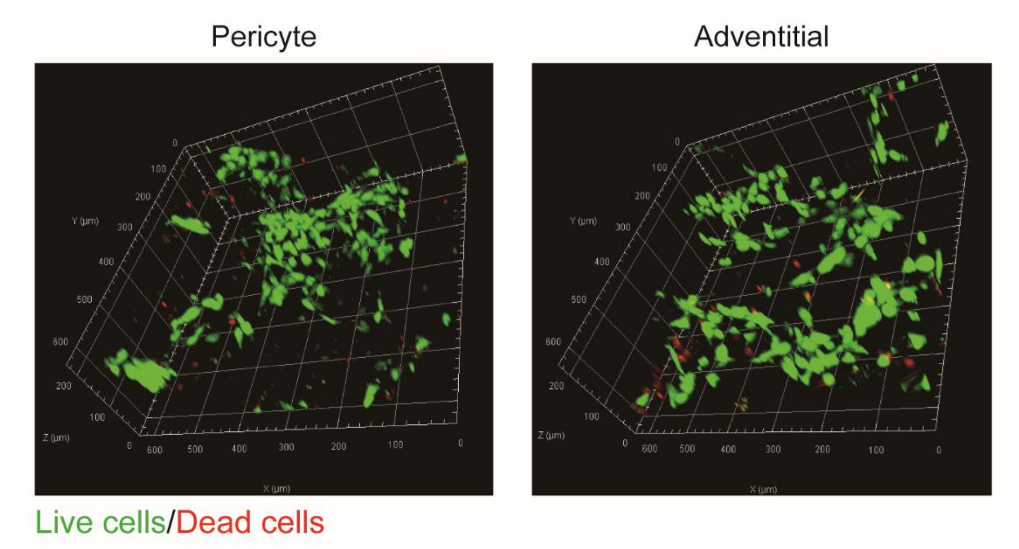

Supplementary Figure 4. CD146$^+$ pericytes or CD34$^+$ adventitial cells were labeled with PKH26 fluorescent dye, followed by engraftment in the calvarial defect model. *In vivo* cell engraftment / persistence was examined after 3 d (top images). Among engrafted cells, patchy Ki67 immunoreactivity was observed among PKH26 labelled cells within the defect site (middle images), and rare TUNEL staining was observed among PKH26 labelled cells (bottom images).

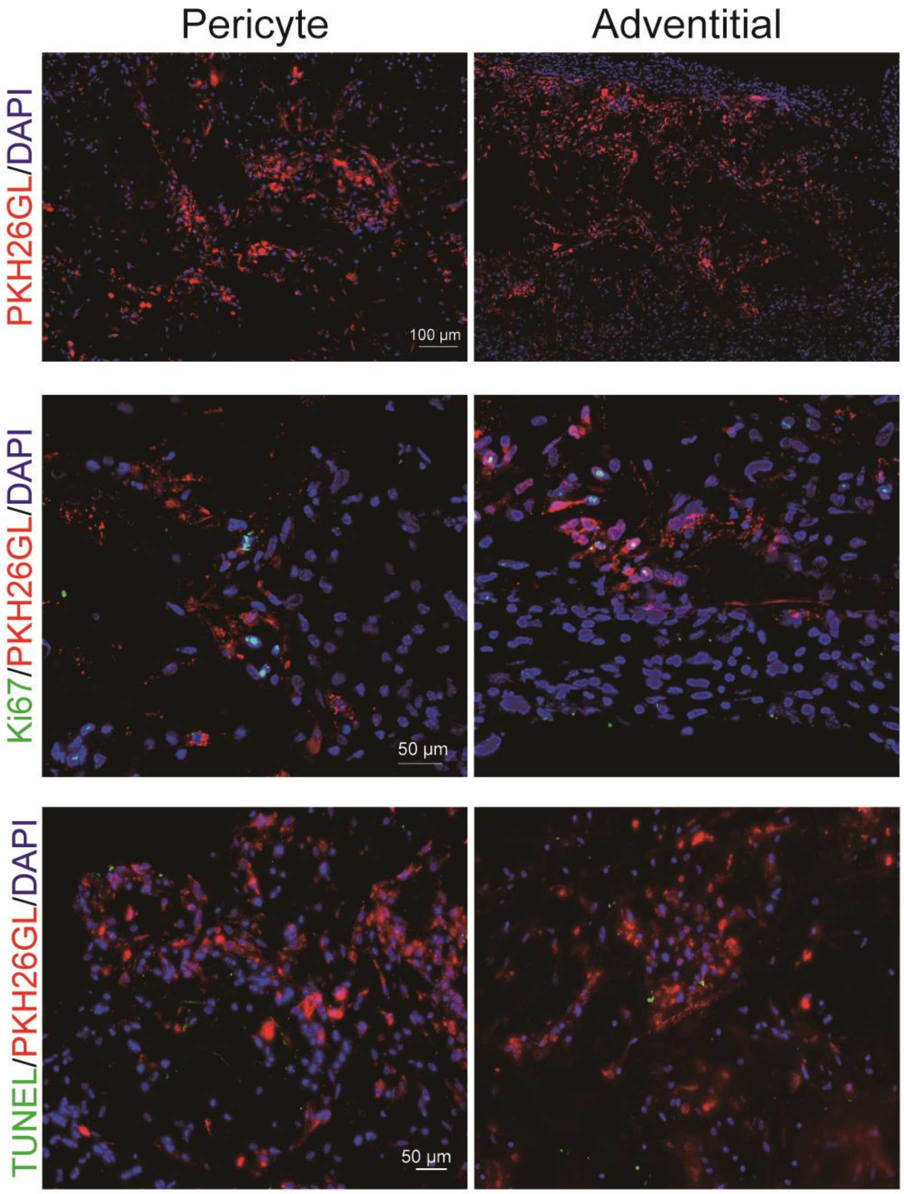

Supplementary Figure 5. Immunohistochemical detection of Human Nuclear Antigen among calvarial defects engrafted with CD146+ pericytes or CD34+ adventitial cells, at eight weeks postoperative. No immunoreactivity was observed at this timepoint.

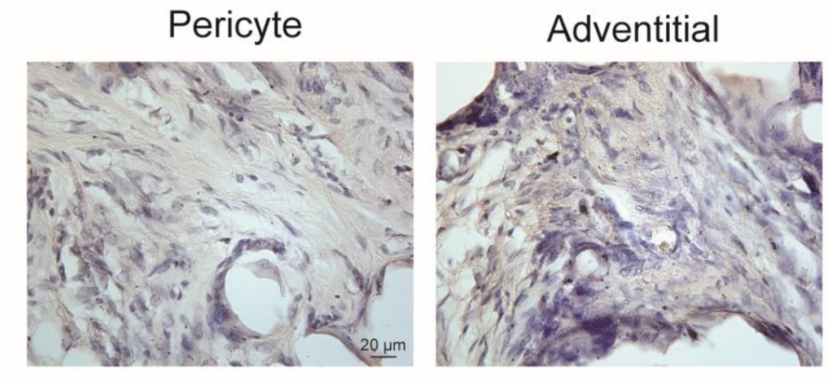

Supplementary Tables

Supplementary Table 1. Antibodies used in fluorescence activated cell sorting

**Antibody**	**Fluorochrome**	**Company-Catalog #**
Mouse Anti-Human CD34	APC	BD Pharmingen-555824
Mouse Anti-Human CD146	FITC	Bio Rad-MCA2141F
Mouse Anti-Human CD45	APC-cy7	BD Pharmingen-557833
Mouse Anti-Human CD31	APC-cy7	Bio Legend-303119

Supplementary Table 2. Summary of treatment groups and animal allocation

Treatment group	Scaffold	Cell #	Defect #
No scaffold	-	-	6
Scaffold alone	HA-coated PLGA	-	4
Pericyte		2.5×10^5 pericytes	4
Adventitial		2.5×10^5 adventitial cells	4
Adventitial + Pericyte		1.25×10^5 adventitial cells + 1.25×10^5 pericytes	4

Supplementary Table 3. Quantitative RT-PCR primer sequences

**Gene**	**Forward**	**Reverse**
GAPDH	5'-CTGGGCTACACTGAGCACC-3'	5'-AAGTGGTCGTTGAGGGCAATG-3'
ALP	5'-ACCACCACGAGAGTGAACCA-3'	5'-CGTTGTCTGAGTACCAGTCCC-5'
RUNX2	5'-TGGTTACTGTCATGGCGGGTA-3'	5'-TCTCAGATCGTTGAACCTTGCTA-3'
COL1A1	5'- GAGGGCCAAGACGAAGACATC-3'	5'- CAGATCACGTCATCGCACAAC-3'
IGFBP1	5'-TTTTACCTGCCAAACTGCAACA-3'	5'-CCCATTCCAAGGGTAGACGC-3'
IGFBP2	5'-GACAATGGCGATGACCACTCA-3'	5'-CAGCTCCTTCATACCCGACTT-3'
MCP1	5'-CAGCCAGATGCAATCAATGCC-3'	5'-TGGAATCCTGAACCCACTTCT-3'
